# Tapered Fiber Bioprobe Based on U-Shaped Fiber Transmission for Immunoassay

**DOI:** 10.3390/bios13100940

**Published:** 2023-10-20

**Authors:** Xinghong Chen, Lei Xiao, Xuejin Li, Duo Yi, Jinghan Zhang, Hao Yuan, Zhiyao Ning, Xueming Hong, Yuzhi Chen

**Affiliations:** 1 School of Physics and Optoelectronic Engineering, Shenzhen University, Shenzhen 518060, China; 2250453009@email.szu.edu.cn (X.C.); 2050453015@email.szu.edu.cn (L.X.); lixuejin@szu.edu.cn (X.L.); yiduo@szu.edu.cn (D.Y.); zhangjinghan@cuhk.edu.cn (J.Z.); 15116090750@163.com (H.Y.); 13824874421@163.com (Z.N.); xmhong@szu.edu.cn (X.H.); 2 Shenzhen Engineering Laboratory for Optical Fiber Sensors and Networks, Shenzhen 518060, China; 3 Shenzhen Key Laboratory of Sensor Technology, Shenzhen 518060, China; 4 Shenzhen Tian’an Zhiyuan Sensor Technology Co., Ltd., Shenzhen 518060, China; 5 School of Science, The Chinese University of Hong Kong, Shenzhen 518172, China

**Keywords:** optical fiber sensors, tapered fiber bioprobes, Mach–Zehnder interference, immunoassay

## Abstract

In this paper, a tapered fiber bioprobe based on Mach–Zehnder interference (MZI) is proposed. To retain the highly sensitive straight-tapered fiber MZI sensing structure, we designed a U-shaped transmission fiber structure for the collection of optical sensing signals to achieve a miniature-insert-probe design. The spectrum responses from the conventional straight-tapered fiber MZI sensor and our proposed sensor were compared and analyzed, and experimental results showed that our proposed sensor not only has the same sensing capability as the straight-tapered fiber sensor, but also has the advantages of being flexible, convenient, and less liquid-consuming, which are attributed to the inserted probe design. The tapered fiber bioprobe obtained a sensitivity of 1611.27 nm/RIU in the refractive index detection range of 1.3326–1.3414. Finally, immunoassays for different concentrations of human immunoglobulin G were achieved with the tapered fiber bioprobe through surface functionalization, and the detection limit was 45 ng/mL. Our tapered fiber bioprobe has the insert-probe advantages of simpleness, convenience, and fast operation. Simultaneously, it is low-cost, highly sensitive, and has a low detection limit, which means it has potential applications in immunoassays and early medical diagnosis.

## 1. Introduction

Biosensors breathe new life into traditional immunoassays. The immunoassay is a highly selective bioanalytical technique that has been widely used and developed in various fields such as clinical diagnosis, drug analysis, biochemical analysis, and environmental monitoring. The main traditional immunoassays are the enzyme-linked immunosorbent assay (ELISA) [1], enzyme immunoassay [2], immunofluorescence assay [3], and lateral flow immunoassay [4]. However, these methods are only suitable for laboratory diagnosis and analysis and require complex instrumentation and demanding testing operations [5]. To simplify immunoassays, the introduction of biosensors is undoubtedly a good choice. Optical fiber biosensors have received wide attention and in-depth research because of their advantages such as fast response, high selectivity, high sensitivity, easy operation, anti-electromagnetic interference, good biocompatibility, miniaturization, and low cost [6]. Through surface functionalization with different biometric components, optical fiber biosensors can detect targets such as antigens [7], nucleic acids [8], cancer markers [9], viruses [10], bacteria [11], etc., which has important applications in the clinical, environmental, and medical fields.

Researchers have designed various types of optical fiber biosensors with different principles, such as surface plasmon resonance (SPR) [12], localized surface plasmon resonance (LSPR) [13], fiber grating [14], distributed fiber [15], and fiber interferometer biosensors [16], through the flexible design of fiber structures and diverse variations in sensing materials. Among them, the optical fiber Mach–Zender interference (MZI) biosensor is one of the most varied-form optical sensors, with forms such as tapered [17], U-shaped [18], and hetero-core structures [19], and so on. To achieve highly sensitive detection, a tapered fiber structure is used to excite a strong evanescent field by reducing the fiber diameter for the enhancement of MZI sensing. In 2021, Liu et al. proposed a label-free biosensor based on S-tapered fiber to achieve an immunoassay for immunoglobulin G (IgG) with a sensitivity of 538.62 nm/RIU and a detection limit (LOD) of 28 ng/mL [20].

In 2022, Luo et al. proposed an optical fiber biosensor based on taper side polishing to achieve highly sensitive detection of human chorionic gonadotropin [21]. To improve the practicality of the tapered fiber biosensor, the insert-probe design is a good choice. In 2023, Qiu et al. proposed a U-shaped biosensor based on tapered fiber to achieve highly sensitive detection of Staphylococcus aureus [22]. However, bending a straight-tapered fiber into a U shape led to the leakage of optical sensing signals and reduced the stability of the sensing structure [23]. If a tapered fiber sensor can be designed as an inserted probe while maintaining the straight-tapered sensing fiber structure, the sensor can not only achieve high-sensitivity biological measurements, but can also meet the requirements of convenient, stable, and highly efficient detection.

In this paper, we propose a tapered fiber bioprobe that maintains the highly sensitive straight-tapered fiber sensing structure, while the insert-probe fiber sensor structure was accomplished through designing a U-shaped transmission fiber. In the detection of gradient refractive index liquids, the tapered fiber bioprobe demonstrated the advantages of fast and convenient detection, obtaining a high sensitivity of 1611.27 nm/RIU in a refractive index range of 1.3326–1.3414. Subsequently, the surface functionalization of the tapered fiber bioprobe was performed by immobilizing antihuman IgG, resulting in a highly reproducible and selective H-IgG assay with a LOD of 45 ng/mL. The sensor has the advantages of high sensitivity, low production cost, and renewability in trace and microspace biological detection.

## 2. Materials and Methods

### 2.1. Materials

Single-mode fiber (Corning Incorporated, Corning, NY, USA, SMF-28) and UV adhesive (ergo,8500) were obtained. Piranha solution was prepared by mixing concentrated sulfuric acid solution (98%) and hydrogen oxide solution (30%) in a volume ratio of 3:1. 3-Aminopropyltriethoxysilane (APTES) was purchased from Shanghai Aladdin Biochemical Technology Co., Ltd. (Shanghai, China). Glutaraldehyde and sodium chloride (NaCl) were purchased from Shanghai Macklin Biochemical Technology Co., Ltd. (Shanghai, China). Phosphate-buffered solution (PBS, pH = 7.4) was purchased from Shenggong Biological Engineering Co., Ltd. (Shanghai, China). Purified human IgG (H-IgG) and antihuman IgG were purchased from Origene Company. Bovine serum albumin (BSA), ovalbumin, and casein were purchased from Sigma-Aldrich Company (St. Louis, MO, USA). All reagents used in this research work were of analytical grade and could be used without further purification. 

### 2.2. Preparation and Working Principle

The proposed sensor was fabricated in two main steps: the first step was to fabricate a straight-tapered fiber sensing structure; the second step was to fabricate a U-shaped fiber transmission structure to form an inserted fiber probe. First, a commercial fiber tapering machine (AFBT-8000, Shandong Kepler Optoelectronics Technology Co., Ltd., Tai’an, China) was used to prepare a straight-tapered fiber. The length of the tapered gradient region was set to 2000 μm, and the length of the tapered waist region was set to 4000 μm with a diameter of 9 μm. Then, a flame lighter (flame temperature 1300 °C) was used to heat the normal transmission fiber region on one side of the tapered region to bend it. The single-mode fiber was bent and shaped into a U-shaped structure when the heating temperature reached the softening temperature. Finally, the finished sensor was fixed on a holder with UV adhesive.

The proposed sensor is composed of the cascade of a straight-tapered fiber structure and a U-shaped transmission fiber structure. The straight-tapered fiber can be considered as an MZI fiber sensor [24]. However, because of the excessive bending of the U-shaped fiber structure, the fundamental mode transmitted in the fiber core excites cladding modes, forming an unavoidable multimode interference (MMI). To simplify the analysis, the sensing process in the straight-tapered fiber structure can be analyzed using MZI theory. When the light transmits to the first tapered region, the high-order cladding mode is excited due to the decrease in fiber diameter, and the cladding mode has a strong evanescent field to sense the external refractive index change. Then, the fundamental mode and the cladding mode transmit through the waist region and are coupled in the second tapered region to excite MZI [25]. The straight-tapered fiber MZI sensing signal is further superimposed with the MMI effect when it passes through the U-shaped transmission fiber structure. Therefore, the final composite interference output intensity is as follows [26]:

(1) $$I=I_{1}+\sum_{i=1}^{n}I_{2}^{i}+2\sum_{i=1}^{n}\sqrt{I_{1}I_{2}^{i}}\cos\Delta \varphi^{i}$$

where $I_{1}$ and $I_{2}^{i}$ are the intensities of the fundamental mode and higher-order cladding modes, respectively. ${\Delta \varphi }^{i}$ is the phase difference between the fundamental mode and other higher-order cladding modes, which can be expressed as

(2) $$\Delta \varphi^{i}=\frac{2\pi \left(\Delta n_{eff}^{t}L_{1}+\Delta n_{eff}^{u}L_{2}\right)}{\lambda }$$

where ${\Delta n}_{\mathrm{eff}}^{t}$ is the effective refractive index difference between the fundamental mode and higher-order cladding modes in the straight-tapered fiber structure, and $L_{1}$ is the length of the tapered sensing region. ${\Delta n}_{\mathrm{eff}}^{u}$ is the effective refractive index difference between MMI modes in the U-shaped transmission fiber structure, $L_{2}$ is the length of the U-shaped transmission fiber, and λ is the input wavelength.

When the refractive index of the external environment changes, ${\Delta n}_{\mathrm{eff}}^{t}$ changes, resulting in a change in ${\Delta \varphi }^{i}$, which further creates a shift in the interference spectrum. The wavelength shift in the interference spectrum (*λ*_dip_) with the change in the external refractive index (*n*_s_) can be expressed as follows [27]:

(3) $$\frac{\partial \lambda_{dip}}{\partial n_{s}}=\frac{\lambda_{dip}}{\Delta n_{eff}^{t}}\frac{\partial \Delta n_{eff}^{t}}{\partial n_{s}}$$

### 2.3. Surface Functionalization

For the immunodetection of H-IgG, surface functionalization of the proposed sensor required the following steps:

(1) Growing hydroxyl groups (-OH). First, the sensor surface was thoroughly cleaned with deionized water, then it was immersed in piranha solution 1 h for growing -OH, and finally cleaned with deionized water again and dried in air.
(2) Silanization treatment. The proposed sensor was immersed in 10% (*v*/*v*) APTES ethanol solution 3 h for silanization treatment and subsequently washed with ethanol and dried.
(3) Activating the aldehyde group (-CHO). The proposed sensor was immersed in 10% (*v*/*v*) glutaraldehyde solution 1 h for -CHO activation and subsequently washed with PBS buffer.
(4) Growing antibodies. The proposed sensor was immersed in antihuman IgG solution at a concentration of 50 μg/mL 90 min for growing antibodies, and then unbound antibodies were washed with PBS buffer [28].
(5) Surface blocking. To prevent nonspecific binding of other substances, the proposed sensor was immersed in 1% (*w*/*v*) BSA solution (in PBS buffer) 1 h to block unoccupied surface-binding sites, then washed with PBS buffer.

### 2.4. Measurement System

In this work, a full broadband light source (BBS, Fiberlake Co., Ltd., Shenzhen, China, WBB400008SFA) was employed as the input light source, and a spectrum analyzer (OSA, Agilent Technologies Ltd., Beijing, China, Agilent 86146B) was used as the output light collector. To facilitate the detection of trace biological samples in microspace, the tapered fiber bioprobe was fixed to the holder for the plug-in detection of samples. During each measurement, the trace sample could easily cover the entire sensing region. Each test was repeated at least three times for error acquisition. Finally, the spectral data were analyzed via computer processing.

## 3. Results and Discussion

### 3.1. Sensing Performance of the Tapered Fiber Bioprobe

**Figure 1:**
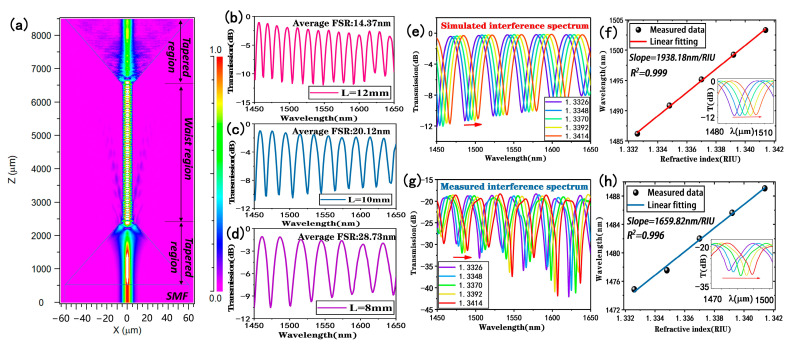
Optimization analysis of a straight-tapered fiber: (**a**) longitudinal optical field energy distribution of a tapered fiber; (**b**–**d**) simulated transmission spectra in air for sensors with tapered lengths of 12 mm, 10 mm, and 8 mm, respectively; (**e**) simulated transmission spectrum of the sensor at different refractive indices at 1450–1650 nm; (**f**) simulated calculated refractive index sensitivity (inset: response of the monitored trough in detecting refractive index); (**g**) experimental transmission spectrum of the sensor at 1450–1650 nm with different refractive indices; (**h**) experimental refractive index sensitivity (inset: response of the monitored trough in detecting refractive index).

**Figure 2:**
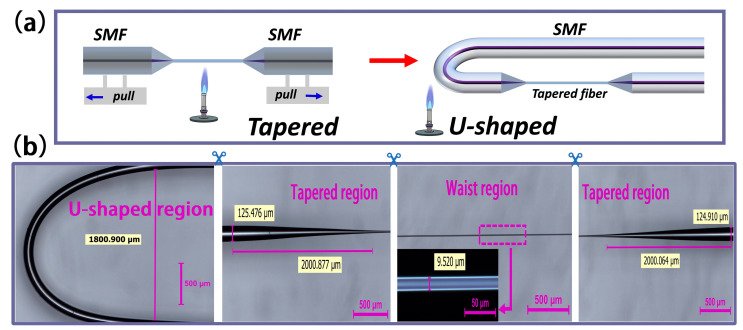
(**a**) Schematic diagram of the sensor fabrication process. (**b**) Optical micrograph of the sensor structure.

In order to study the sensing performance of the tapered fiber bioprobe, the structural parameters of the tapered fiber were first simulated and optimized. The beam propagation method of Rsoft software (version 2018.12) was used to simulate a single-mode fiber with two tapering regions of about 2000 μm in length and a uniform taper-waist region of 4000 μm in length and a diameter of 9 μm to obtain the longitudinal light field distribution in air when the wavelength of the incident light was 1550 nm, as shown in Figure 1a. It can be seen that the optical field energy in the core of a single-mode fiber leaks into the cladding as the diameter of the tapered region decreases, thus exciting higher-order cladding modes and transmitting them to the next tapered region for coupling. Keeping the other parameters constant, the length of the waist region was varied and the transmission spectra in air were obtained via simulation for lengths of 12 mm, 10 mm, and 8 mm, respectively, as shown in Figure 2b–d. It was found that the interference transmission spectrum of the tapered fiber in air had a sinusoidal waveform and the free spectral range (FSR) of the transmission spectrum gradually increased with the decreasing sensing lengths, which were 14.37 nm, 20.12 nm, and 28.73 nm, respectively. According to the formula of FSR = λ^2^/∆neff·L, FSR is inversely proportional to L when λ and ∆neff are certain. Considering that the sensor performs refractive index measurements, a small FSR may result in wavelength drift beyond the period range. In addition, it has been demonstrated in our previous work that a tapered fiber with a waist diameter of around 10 μm can possess a better surface swift field [29]. Thus, we chose the fiber with a length of 8 mm and taper-waist diameter of 9 μm for the next study.

In order to simulate and analyze the response of the straight-tapered fiber in detecting the external environment, the response transmission spectrum was calculated within the refractive index range of 1.3326–1.3414 and the wavelength range of 1450–1650 nm, as shown in Figure 1e. As can be seen from Figure 1e, the transmission spectrum shows a red shift as the refractive index increases. Figure 1f shows a linear fit analysis of the trough of the transmission spectrum near the wavelength of 1500 nm, and the slope of the linear relationship is the sensitivity, which in turn is calculated to give a refractive index sensitivity of 1938.18 nm/RIU with a linear fitting of 0.999. Then, we fabricated a straight-tapered fiber sensing structure with the optimized parameters obtained from the above simulation to verify its refractive index sensing performance. NaCl solutions with refractive indices of 1.3326, 1.3348, 1.3370, 1.3392, and 1.3414 were configured, and their refractive indices were calibrated with an Abbe refractometer (INESA Instrumental Physics and Optics Co., Ltd., Shanghai, China, WAY-2S) at room temperature. Figure 1g shows the response spectrum of experimentally prepared tapered fibers at different refractive indices, and the transmission spectra show a red shift in wavelength, which is consistent with the analysis of the simulation calculations. The results shown in Figure 1h were obtained by monitoring the dip shift near the wavelength of 1500 nm. The refractive index sensitivity of the tapered fiber was 1659.82 nm/RIU, and the response wavelength and refractive index showed a good linear relationship with a linear fitting of 0.996. The difference between the simulation and experimental results may be due to uncontrollable factors generated during the experimental fabrication of the tapered fiber.

**Figure 3:**
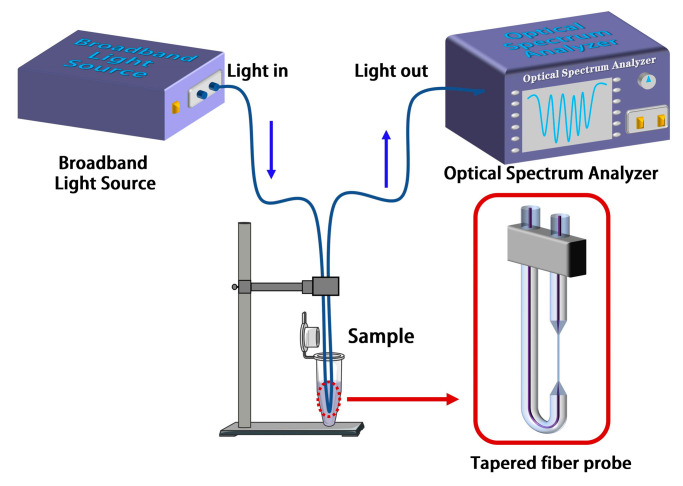
Schematic diagram of the experimental setup of the tapered fiber bioprobe.

Figure 2a shows the fabrication process of a tapered fiber bioprobe, divided into a straight-tapered fiber structure and a U-shaped transmission fiber structure. The micrograph of the fabricated bioprobe shows that the length of the tapered region is about 2000 μm, and that of the 9.52 μm diameter waist region is 4000 μm (Figure 2b). Additionally, in order to not reduce the mechanical strength of the straight-tapered fiber structure for the subsequent fabrication of the U-shaped fiber structure, the 9.52 μm waist diameter was chosen. The smaller the bend radius of the U-shaped fiber structure, the more favorable the miniaturization of the sensor, and the operable limit bend radius is about 0.9 mm in this case. The U-shaped fiber design changes the on-line transmission fiber sensing system into a plug-in fiber probe detection system (Figure 3), facilitating the measurement of biological samples.

**Figure 4:**
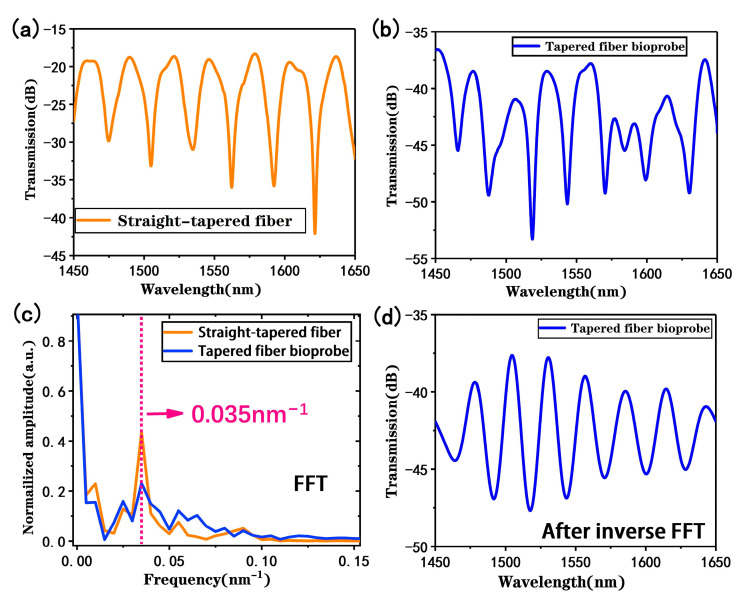
(**a**) Transmission spectrum of a straight-tapered MZI sensor in measuring deionized water; (**b**) transmission spectrum of the proposed tapered fiber bioprobe in measuring deionized water; (**c**) spatial frequency distribution of straight-tapered fiber and proposed tapered fiber bioprobes; (**d**) transmission spectrum after IFFT transformation.

In our design, the straight-tapered fiber stimulates the main MZI sensing signal, while the U-shaped fiber is fabricated for the plug-in probe design. However, both structures bring mode leakages and loss of transmitted light on sensing [30]. Therefore, the spectral signals of the straight-tapered fiber before and after cascading the U-shaped fiber were comparatively analyzed, as shown in Figure 4a, where it can be observed that the interference fringes in the transmission spectrum of the taper fiber have a better periodicity and fringe visibility, and the insertion loss is about 18 dB. Subsequently, a U-shaped fiber structure was fabricated on one side of the straight-tapered fiber MZI sensor, and Figure 4b displays its spectral signal. It can be observed that the interference spectrum becomes complex, which is attributed to the superposition of multiple interference modes. And the interference fringes still have good fringe visibility; the increasing insertion loss of 37 dB is attributed to the U-shaped fiber bending. The spatial frequency distribution shown in Figure 4c was obtained via fast Fourier transform (FFT), revealing that the main interference frequency is 0.035 nm^−1^, and other weaker frequency peaks are caused by the interference between the fiber core mode and different order cladding modes in the tapered structure. After cascading the U-shaped structure, the MZI signal stimulated by the straight-tapered fiber still exists (not submerged in the MMI signal caused by the U-shaped fiber), and the main interference frequency is still 0.035 nm^−1^. However, multimode interferometric signals are a superposition of multiple sinusoidal waveforms, and the monitored dips may be caused by other interferometric modes, so it is difficult to accurately detect targets through the dominant interferometric frequency. Thus, the main interference frequencies in the spectrogram are filtered and processed via inverse fast Fourier transform (IFFT) to obtain the two-beam interference spectrum shown in Figure 4d. Therefore, our tapered fiber bioprobe that combines a straight-tapered fiber MZI sensor and a U-shaped fiber MMI sensor can still work in MZI mode by extracting the main interference frequency.

**Figure 5:**
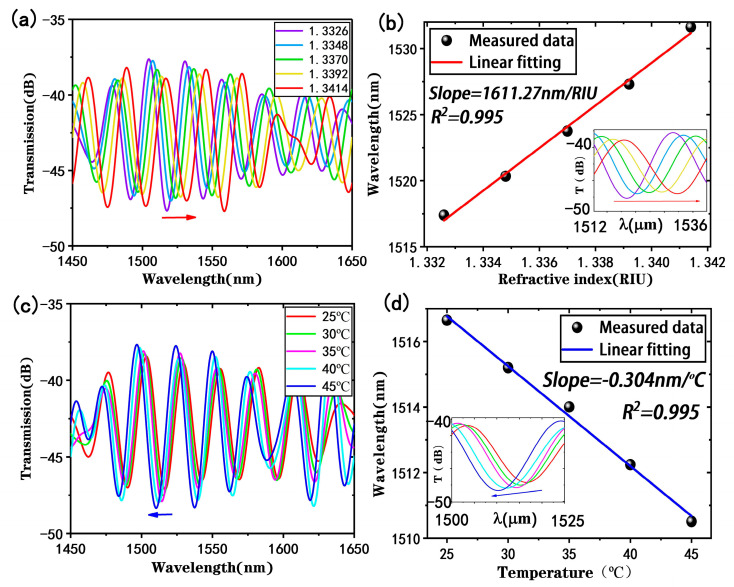
Refractive indices and temperature responses of the tapered fiber bioprobe: (**a**) transmission spectra at different refractive indices; (**b**) refractive index sensitivity (inset: response of monitored dip in detecting refractive index); (**c**) transmission spectra at different temperatures; (**d**) temperature sensitivity (inset: response of monitored dip in detecting temperature).

To study the performance of the proposed tapered fiber bioprobe, the sensor was used to detect NaCl solutions with a refractive index range of 1.3326–1.3414. Figure 5a shows the variation in the response of the transmission spectrum of the sensing probe in detecting different refractive indices after filtering and IFFT processing, and it can be observed that the transmission spectrum exhibits a red shift as the refractive index increases, which is caused by the increasing ∆φ^i^. By monitoring the dip shift where the stripe contrast was most pronounced, the refractive index sensitivity of the proposed bioprobe was found to be 1611.27 nm/RIU, as shown in the linear fitting analysis in Figure 5b. The wavelength has a good linear relationship with the detected refractive index, with a linear fitting of 0.995. The refractive index sensitivity of the proposed bioprobe is slightly reduced compared with the straight-tapered fiber MZI sensor, which may be due to uncontrolled losses from the U-shaped fabrication.

In the actual measurements, temperature fluctuations can affect the detection of refractive indices and even the detection of biomolecules. Therefore, the temperature sensing performance of the tapered fiber bioprobe was evaluated. Figure 5c shows the transmission spectrum in a temperature range of 25–45 °C, indicating a blue shift with increasing temperature in the interference spectrum. According to the linear fitting analysis results in Figure 5d, the valley wavelength has a good linear relationship with temperature, with a linear fit of 0.995 and a temperature sensitivity of −0.304 nm/°C. Since the subsequent biological experiments were conducted at a constant temperature of 25 °C, the temperature effect could be ignored.

### 3.2. Immunoassay Performed with the Tapered Fiber Bioprobe

**Figure 6:**
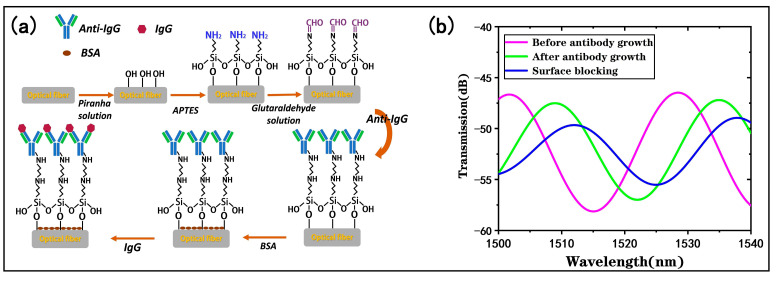
Biological modifications of the tapered fiber bioprobe: (**a**) immunofunctionalization process; (**b**) changes in transmission spectrum of sensor surfaces after antibody modification and surface blocking.

To achieve highly sensitive and selective target biomolecule detection, surface functionalization of the tapered fiber bioprobe is necessary [31]. The steps of the surface functionalization are shown in Figure 6a, and the following experiments were performed at a constant temperature (25 °C).

By monitoring the transmission spectrum of the tapered fiber bioprobe, optical responses before and after the antibody immobilization can be determined. Figure 6b shows the transmission spectrum of the bioprobe in PBS buffer before and after the antibody modification, and it can be found that the antibody modification shifted the transmission spectrum by 7.1 nm to the longer wavelength, which is attributed to the antibody modification on the bioprobe surface through -CHO. The covalent binding of the antibody causes a refractive index change on the bioprobe surface. The results indicate that the antibody was effectively immobilized on the bioprobe surface. By monitoring the transmission spectrum of the antibody-modified probe in PBS buffer after surface blocking, it can be found that the blocking of the residual nonspecific binding sites on the surface of the sensing probe is effectively accomplished.

**Figure 7:**
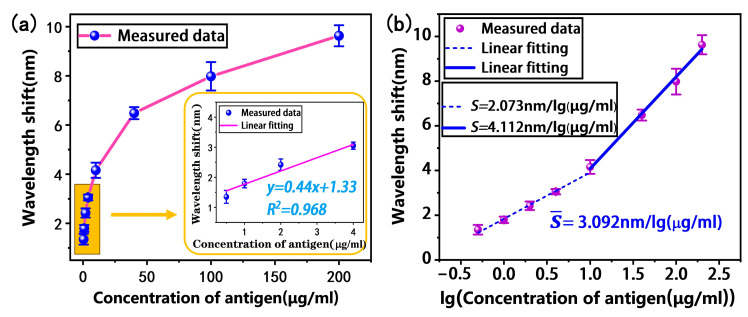
Target antigen H-IgG detection results: (**a**) wavelength responses at different concentrations (inset: linear fitting in the low-concentration range); (**b**) wavelength responses at different logarithmic concentrations.

We evaluated the immunoassay performance of the tapered fiber bioprobe. First, the antibody-modified tapered fiber bioprobe was used to detect H-IgG at different concentrations (0.5, 1, 2, 4, 10, 40, 100, 200 μg/mL) for 30 min. Subsequently, the bioprobe was washed with PBS buffer to remove the unbound H-IgG. Finally, the transmission spectrum shift was monitored in the PBS buffer. The results in Figure 7a show a saturation curve with increasing antigen concentration. The specific antigen–antibody binding increases the local refractive index on the bioprobe surface, thus causing a shift in the transmission spectrum. The inset in Figure 7a shows the results of the linear fitting analysis within the low-concentration H-IgG range of 0.5–4 μg/mL, with a detection sensitivity of 0.44 nm/(μg/mL) and a linear fitting of 0.968. Further, the limit of detection is one of the most crucial performance parameters for biosensors; the LOD of the bioprobe is expressed as follows [32]:

(4) $$LOD=\frac{\Delta \lambda }{S}$$

Here, ∆λ = 0.02 nm is the wavelength resolution of our spectrometer and S is the detection sensitivity of the sensor; thus, the LOD is calculated to be 45 ng/mL.

To facilitate the fitting analysis, the logarithmic concentration was taken to obtain the relationship between the wavelength shift and logarithmic concentration, as shown in Figure 7b. It can be found that the wavelength shift in the low- and high-concentration intervals shows a two-band linear distribution with respect to the logarithmic concentration. The linear fitting analysis shows that the average sensitivity of the tapered fiber bioprobe in the H-IgG concentration range of 0.5–200 μg/mL is 3.092 nm/lg (μg/mL).

**Figure 8:**
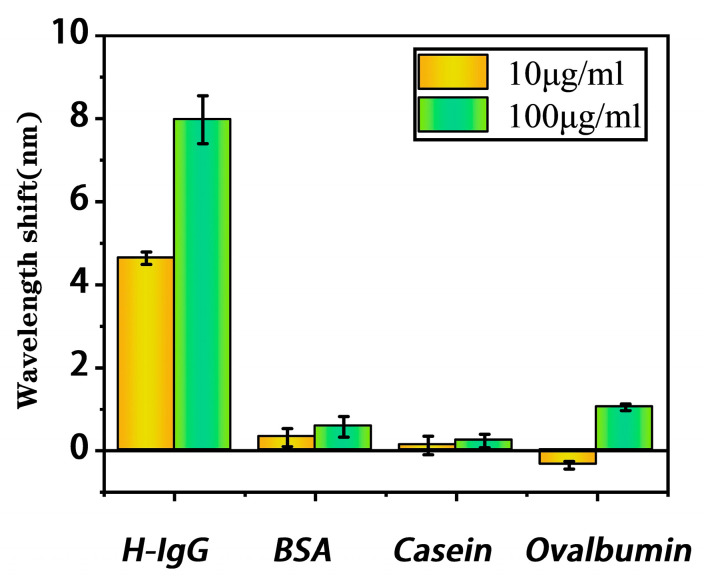
Selectivity of the proposed bioprobe.

Sample solutions of H-IgG, BSA, ovalbumin, and casein, with high and low concentrations (10 μg/mL and 100 μg/mL), were measured with the proposed bioprobe. The selectivity results in Figure 8 indicate that the proposed bioprobe has a significant response to H-IgG and no response to other molecules. The proposed bioprobe has the ability to specifically detect H-IgG.

Our tapered fiber bioprobe has the advantage of an insert-probe for effortless detection and is distinguished by its low cost, high sensitivity, and miniaturization. The surface of the bioprobe can be uniformly modified for one-time mass production through functionalization, while the detection system can be further minimized, enabling the manufacture of point-of-care (POC) devices.

Finally, a comparison of different types of fiber optic IgG biosensors is listed in Table 1.

## 4. Conclusions

In summary, we proposed and fabricated a tapered fiber bioprobe for the immunoassay of H-IgG. To retain the highly sensitive straight-tapered fiber MZI sensing structure, we designed a U-shaped transmission fiber structure for the collection of optical sensing signals to achieve a miniature-insert-probe design. The proposed bioprobe achieved a high sensitivity of 1611.27 nm/RIU in the refractive index range of 1.3326–1.3414. After surface functionalization, the proposed bioprobe realized the immunoassay of H-IgG with an average sensitivity of 3.092 nm/lg (μg/mL) and a LOD of 45 ng/mL. The portable immunosensor developed in this study has potential applications in immunoassays and early medical diagnosis due to its high sensitivity, low production cost, plug-in microprobe structure, and convenient use.

## Figures and Tables

**Table 1:** Performance of different types of fiber optic IgG biosensors.

Sensor Type	Detection Range	LOD	Expense	Practicality	Reference
Fiber optic SPR	10–100 μg/mL	3.4 μg/mL	High	Sample-injection	[33]
Etched SMS fiber	4 mg/L–200 mg/L	0.2 mg/L	Moderate	Sample-injection	[34]
Fiber optic SPR	0–80 μg/mL	0.104 μg/mL	High	Insert-probe	[35]
Elliptical core helical Intermediate period Fiber grating	0–100 μg/mL	4.7 μg/mL	High	Sample-injection	[36]
S-tapered fiber	--	1 μg/mL	Low	Sample-injection	[37]
Tapered fiber bioprobe	0–200 μg/mL	45 ng/mL	Low	Insert-probe	This work

## Data Availability

Not applicable.
